# Negativity towards negative results: a discussion of the disconnect between scientific worth and scientific culture

**DOI:** 10.1242/dmm.015123

**Published:** 2014-02

**Authors:** Natalie Matosin, Elisabeth Frank, Martin Engel, Jeremy S. Lum, Kelly A. Newell

**Affiliations:** 1 Faculty of Science, Medicine and Health and Illawarra Health and Medical Research Institute, University of Wollongong, NSW 2522, Australia.; 2 Schizophrenia Research Institute, Sydney, NSW 2010, Australia.

**Keywords:** Negative findings, Negative results, Null hypothesis

## Abstract

Science is often romanticised as a flawless system of knowledge building, where scientists work together to systematically find answers. In reality, this is not always the case. Dissemination of results are straightforward when the findings are positive, but what happens when you obtain results that support the null hypothesis, or do not fit with the current scientific thinking? In this Editorial, we discuss the issues surrounding publication bias and the difficulty in communicating negative results. Negative findings are a valuable component of the scientific literature because they force us to critically evaluate and validate our current thinking, and fundamentally move us towards unabridged science.

“What gets us into trouble is not what we don’t know, it’s what we know for sure that just ain’t so.” – Mark Twain.

## The impact of negative findings

Increasingly, there is pressure on scientists to choose investigative avenues that result in high-impact knowledge. This challenge has, in many cases, swayed scientists to pursue paths of investigation that are not necessarily logical or hypothesis-driven. Rather than approaching a research question in a systematic manner, it seems that scientists are encouraged to pursue non-linear lines of investigation in search of significance, and many that have the luxury are known to tuck away negative findings (the ‘file-drawer’ effect) and focus on their positive outcomes (Scargle, 1999). This behaviour likely stems from an ever-heightening hurdle that scientists need to jump: high publication output with a high citation rate in order to win competitive grants to drive their research, move up the rungs and pay the bills.

Published a few years ago in *PLoS ONE*, Daniele Fanelli states that “Papers are less likely to be published and to be cited if they report ‘negative’ results” (Fanelli, 2010). Because scientists are involuntarily finding themselves engaged in competition for positions and funding, many are choosing not to proceed with their non-significant findings (those that support the null hypothesis) that yield less scientific interest and fewer citations. Consequently, the amount of non-significant data reported is progressively declining (Fanelli, 2012). Although it could be argued that this is due to an increasing quality of science, it is more likely attributable to the selectiveness of ‘high impact’ journals that, in our opinion, might as well have a bold statement in the submission form: **negative results are not accepted**. However, there seems to be a gap between results that are positive and results that are high impact. Logically there is no connection, but it seems scientific culture assumes that they are analogous. Why aren’t negative results considered to be of the same value?

Historically, the noblest aspect of science is its supposed transparency in presenting all sides of a story. In theory, scientific principles are always under reconsideration and, indeed, there are occasions (which we will subsequently discuss) where new evidence has refuted old hypotheses and impacted on current scientific thinking. This seems reasonable, but is perhaps easier said than done. One of the most prominent examples was provided by London-based research doctor Andrew Wakefield, who, together with 12 co-authors, published the radical finding that child vaccination (specifically the MMR vaccine) increases the incidence of autism (Wakefield et al., 1998). The now infamous paper, which appeared in *The Lancet*, triggered widespread panic that led to a decade-long decrease in child immunisation. In spite of the 13 studies with convincing negative results published between 1998 and the article retraction in 2010 (see table 1 of Gerber and Offit, 2009), support against Wakefield’s claims failed to gain the same level of attention as the original study, evidenced by a rise in morbidity and mortality of preventable diseases, including measles, mumps and rubella, during this time (see Gerber and Offit, 2009).

The process of transitioning between paradigms of current scientific thinking particularly fascinated the famous American physicist and philosopher Thomas Kuhn. Kuhn’s writings propose that once the body of evidence for the competing paradigm overtakes the evidence in support of the dominant paradigm, then scientists will easily switch allegiance (Kuhn, 1970). However, there are greater forces influencing this process, according to the theory that humans have an inbuilt need to support the status quo, and therefore have an innate difficulty in overriding preexisting beliefs (Jost and Hunyady, 2003). When faced with the challenges of opposing current views with new research, our own cognitive bias makes it difficult to muster up the strength to fight for the paradigm shift, especially because negative results are often associated with flawed or poorly designed studies, and thus might be viewed as a negative reflection on the scientist. Therefore, negative results are an inconvenient truth, and ignoring the inconsistent results would only be human.

## Correcting the literature: an uphill battle

Although a search of recent literature will turn up periodic examples in which current research rejects previously published ideas, the difficulty that arises in attempting to do so, particularly on a large scale, is greatly ignored. Extreme difficulties in correcting the literature have been experienced by many, but discussed openly by few. Consultant cardiologist Dr Peter Wilmshurst was particularly proactive in discussing this topic and his own experiences. Wilmshurst dedicated himself for two decades to disseminating his negative findings to reverse the aftermath of a drug trial regarding the heart-contractility drug amrinone. The original article, published in *The New England Journal of Medicine* (Benotti et al., 1978), stated that amrinone increased heart contractility in a small clinical trial. Wilmshurst’s subsequent studies, by contrast, repeatedly showed that “although amrinone increased the strength of contraction of normal heart muscle, it did not affect contractility in patients with heart failure” (Wilmshurst, 2003). Interestingly, in spite of Wilmshurst’s efforts to disseminate these findings and push for article retraction, he remains unsuccessful.

Australian cell biologist Professor David Vaux recently published an essay regarding his own attempts to refute research and retract his work from arguably the most influential scientific journal, *Nature* (Vaux, 2013). In 1995, Vaux was invited to peer-review a paper for the journal and was extremely excited by the results that proposed a mechanism to overcome the rejection of transplanted tissue (Bellgrau et al., 1995). This paradigm-changing data influenced him to both write a ‘News and Views’ piece, published in the same issue of *Nature* (Vaux, 1995), as well as directed his own laboratory’s future research. However, over the next years, the Vaux lab repeatedly failed to replicate these experimental outcomes. Despite *Nature*’s policies to publish work that refutes data in their publications, Vaux’s findings were ultimately rejected, and later received the same fate from *Nature Medicine*. Vaux writes, “Little did we know that instead of providing an answer to transplant rejection, these experiments would teach us a great deal about editorial practices and the difficulty of correcting errors once they appear in the literature” (Vaux, 2013). After 2 years, Vaux finally succeeded in publishing his negative findings in *PNAS* (Allison et al., 1997) and retracted his *Nature* ‘News and Views’ piece.

Science is, by its nature, a collaborative discipline, and one of the principal reasons why we should report negative results is so our colleagues do not waste their time and resources repeating our findings. Should Vaux not have so forcefully pushed for his negative findings to be published and for retraction of his commentary, there is no doubt that scientists would still be pursuing research in this direction, wasting valuable time and resources. Interestingly, shortly after Vaux was granted the retraction, *Nature Medicine* published a paper in support of the negative results despite originally rejecting Vaux’s negative findings (Kang et al., 1997). Now, all the findings – positive and negative – have been disseminated and all in the field are entirely informed. Unfortunately, this is not always the case.

## Examples from psychiatric research

At a recent conference, two colleagues discovered that they had both unsuccessfully attempted to alter depression-like behaviour in the CD1 mouse strain (a widely used animal model for toxicology studies) with a variety of classical antipsychotics. These findings were surprising in light of the many studies demonstrating efficacy of antipsychotic drugs in different experimental models. Realising that this had occurred in two separate labs, they considered that this might not have been a lack of experiments performed using CD1 mice, but rather a lack of publications on negative findings. They have since corroborated their findings with others. Because the results were unpublished, research groups had continued to follow the same lines of thought and the same paths of investigation, only to all fail in the same way, ultimately wasting time and resources. To our knowledge, these results remain unpublished.

Fundamentally, not publishing the inefficiency of antipsychotic drugs in this animal model could be detrimental to the progression of science; however, one can understand why it was not pursued. The time and effort required to construct the paper and survive the peer-review process is not outweighed by the benefits. Other than ‘for the greater good of science’, this information is not considered high-impact knowledge, and will not result in a highly cited paper. When time is money, and our research output is judged based on impact and citations, why waste the time? In our view, negative results are just as useful as positive findings, but, unfortunately, they do not attract the same citations (Fanelli, 2010).

Pessimism surrounding negative results is obviously a problem, but how are we going to reverse the anti-negative-finding culture? As proposed by Lieberman and Cunningham (Lieberman and Cunningham, 2009), “Perhaps a lab should have to correct for the total number of published results in a given year”. The authors suggest that researchers should be obligated to retract their previous works throughout the progression of their career as they “…[find] that [their] previous tests in old papers are no longer significant in light of their success and, ironically, [their] contribution to the field” (Lieberman and Cunningham, 2009). The view of these authors is that the pressure for positive outcomes is simply putting the scientific community under unnecessary burden and decreases the overall research quality. Negative findings are fundamental to science: they encourage good scientific practice, teach us to critically analyse our pre-existing thoughts and direct new avenues of research. However, while the current scientific culture continues to favour positive findings, negative results will continue to face criticism.

This was the case within our own group when we attempted to disseminate ‘negative’ results. We focus on metabotropic glutamate receptors (mGluRs), which have been implicated in the pathology of schizophrenia and are proposed to be targets of antipsychotic intervention. Despite the large amount of interest and money being poured into the development of mGluR-based therapeutics to treat schizophrenia, the status of these receptors in the pathological state is largely unknown. Before our group embarked on this investigative path, it was unclear whether mGluR protein expression was affected in the pathological states that the novel therapeutics aimed to treat, so examination of mGluR protein expression was a crucial and linear line of investigation. Our findings showed that mGluR2/3 (Frank et al., 2011) and mGluR5 (Matosin et al., 2013) expression was not affected in the pathophysiology of schizophrenia in one of the largest post-mortem human cohorts to date. In the follow-up study, we showed that total mGluR2/3 and mGluR5 expression was unchanged in the cortex of 52 patients with schizophrenia across three independent cohorts compared with controls (our unpublished observations). If you approach these results from the perspective of uncovering the underlying cause of schizophrenia, then yes, these results have little impact. However, in light of the convincing preclinical studies that show promising therapeutic benefits of mGluR-based drugs in schizophrenia-relevant paradigms, our results have interesting translational consequences: if mGluR expression was overly reduced in the pathological state, then one would anticipate problems with the efficacy of these novel drugs *in vivo*. Although our findings were published in journals that recognise the value in reporting negative findings, we have found that members of the wider scientific community are less accepting. Indeed, we particularly faced resistance at scientific conferences when disseminating our evidence as we were criticised for overstating our findings. It makes one wonder, why is a negative finding viewed as such a bad thing? The notion of a negative finding does seem more philosophical than practical. A negative result is in response to a positive question. If you rephrased to a negative question, does that mean you have a positive finding? These negative findings were integral to our group, positively directing our group’s research and encouraging us to look beneath the surface, where we have since uncovered crucially relevant mechanisms.

## Negating the negativity

The revolt against publication bias has sparked a movement in which some have attempted to reverse the pessimism towards negative results. For example, a group of journals have been specifically created to publish the ‘rejects’ (*Journal of Negative Results in Biomedicine*, *The All Results Journals*, *Journal of Articles in Support Of the Null Hypothesis*…and there are more), and new ways to provide access to negative data have emerged (e.g. https://pubpeer.com/). Negative findings are also increasingly represented in broad-scope journals such as *Disease Models & Mechanisms* and *PLoS ONE*. Even so, new journals launched with the specific scope of publishing negative findings often do not attract as many papers, demonstrating that it is the underlying scientific culture that requires change and not only the journal policies.

Publication bias is a common theme in the history of science, and it still remains an issue. This is encapsulated in a piece of commentary published in *Nature*: “…negative findings are still a low priority for publication, so we need to find ways to make publishing them more attractive” (O’Hara, 2011). Negative findings can have positive outcomes, and positive results do not equate to productive science. A reader commented online in response to the points raised by O’Hara: “Imagine a meticulously edited, online-only journal publishing negative results of the highest quality with controversial or paradigm-shifting impact. *Nature Negatives*” (O’Hara, 2011). Negative results are considered to be taboo, but they can still have extensive implications that are worthy of publication and, as such, real clinical relevance that can be translated to other related research fields.

So, although the current scientific culture assumes that negative results are not worthy of attention, here we present another perspective. Sharing negative results does not mean making a story out of dust, that the results are less significant (excuse the pun), or that the results should go unpublished. It means that the direction of scientific research should not be determined by the pressure to win the ‘significance lottery’, but rather systematic, hypothesis-driven attempts to fill holes in our knowledge. At the core, it is our duty as scientists to both: (1) publish all data, no matter what the outcome, because a negative finding is still an important finding; and (2) have a hypothesis to explain the finding. If the experiment has been performed to plan, the data has not been manipulated or pulled out of context and there is compiled evidence of a negative result, then it is our duty to provide an explanation as to why we are seeing what we are seeing. Only by truly rethinking the current scientific culture, which clearly favours positive findings, will negative results be esteemed for their entire value. Only then can we work towards an improved scientific paradigm.
